# A role for the age‐dependent loss of α(E)‐catenin in regulation of N‐cadherin expression and cell migration

**DOI:** 10.14814/phy2.12039

**Published:** 2014-06-11

**Authors:** LaNita A. Nichols, Elizabeth A. Grunz‐Borgmann, Xinhui Wang, Alan R. Parrish

**Affiliations:** 1 Medical Pharmacology and Physiology, School of Medicine, University of Missouri, Columbia, Missouri

**Keywords:** Aging, migration, N‐cadherin, wound repair, α‐catenin

## Abstract

The aging kidney has a decreased ability to repair following acute kidney injury. Previous studies from our laboratory have demonstrated a loss in α‐catenin expression in the aging rat kidney. We hypothesize that loss of α‐catenin expression in tubular epithelial cells may induce changes that result in a decreased repair capacity. In these studies, we demonstrate that decreased α‐catenin protein expression is detectable as early as 20 months of age in male Fischer 344 rats. Protein loss is also observed in aged nonhuman primate kidneys, suggesting that this is not a species‐specific response. In an effort to elucidate alterations due to the loss of α‐catenin, we generated NRK‐52E cell lines with stable knockdown of α(E)‐catenin (C2 cells). Interestingly, C2 cells had decreased expression of N‐cadherin, decreased cell–cell adhesion, and increased monolayer permeability. C2 had deficits in wound repair, due to alterations in cell migration. Analysis of gene expression in the migrating control cells indicated that expression of N‐cadherin and N‐CAM was increased during repair. In migrating C2 cells, expression of N‐CAM was also increased, but the expression of N‐cadherin was not upregulated. Importantly, a blocking antibody against N‐cadherin inhibited repair in NRK‐52E cells, suggesting an important role in repair. Taken together, these data suggest that loss of α‐catenin, and the subsequent downregulation of N‐cadherin expression, is a mechanism underlying the decreased migration of tubular epithelial cells that contributes to the inability of the aging kidney to repair following injury.

## Abstract (precis)

Aging is associated with loss of α‐catenin and N‐cadherin expression in the kidney. In these studies, we demonstrate that α‐catenin regulates, in part, N‐cadherin expression and migration in tubular epithelial cells.

## Introduction

A relationship between acute kidney injury (AKI) and the elderly has long been recognized. In 1972, studies suggested that 23.8% of patients with acute renal failure were >60 years of age (Stott et al. 1972) and the following year it was shown that 13.7% were >70 (Kumar et al. 1973). In 1987, these percentages had risen to 76% and 46%, respectively (Rosenfeld et al. 1987). Importantly, clinical data suggest that the aging kidney has a decreased repair capacity following acute injury. Schmitt et al. (2008a) examined data from 17 studies of AKI and found that a higher percentage of surviving elderly (>65 years) patients did not recover renal function as compared to younger patients. Analogous results have been seen in animal models. Cantley and coworkers demonstrated that Zag (zinc‐a(2)‐glycoprotein), an inhibitor of epithelial cell proliferation, is elevated (6.4‐fold) in proximal tubular epithelial cells from aging mice (19–24 months; Schmitt et al. 2008b). Overexpression of Zag decreased proliferation of proximal tubular epithelial cells in vitro and, importantly, knockdown of Zag in the kidneys of aged mice using siRNA increased proliferation following ischemia–reperfusion injury in vivo.

Renal repair after injury is a complex process involving the correct spatial and temporal sequencing of signaling pathways. A number of growth factors including hepatocyte growth factor, epidermal growth factor, heparin binding epidermal growth factor, transforming growth factor‐α, and insulin‐like growth factor‐1 are increased after injury and are important in the proliferative component of tissue repair (Hammerman 1998; Schena 1998). Recent studies have focused on the cell types responsible for tubular epithelial repair; however, recent data from elegant lineage‐tracing mouse models indicate that the differentiated tubular epithelial cells are responsible for repair as opposed to a population of intratubular stem cells (Kusaba et al. 2014). While proliferation is important in the repair of the kidney following injury, migration of tubular epithelial cells also plays a critical role in the recovery following injury (Cuppage and Tate 1967; Haagsma and Pound 1980; Kartha and Toback 1992; Toback 1992).

The cadherin gene superfamily encodes for transmembrane proteins that regulate calcium‐dependent cell–cell adhesion (Yamada et al. 2005). A functional cadherin adhesion complex requires interaction with cytoplasmic proteins, the catenins. α‐catenin does not directly bind to cadherins; rather, it interacts with the cytoplasmic domain of cadherins via β‐ or γ‐catenin. p120‐catenin binds to the cadherin cytoplasmic domain, and shares sequence homology with β‐ and γ‐catenin, but does not bind α‐catenin. There are several forms of α‐catenin; α(E)‐catenin is epithelial; α(N)‐catenin is neuronal; α(T)‐catenin is expressed in heart and testes, while α‐catulin is a catenin‐like molecule (Kobielak and Fuchs 2004). Previous studies in our laboratory have shown a loss of α‐catenin and N‐cadherin expression in the proximal tubular epithelium in aged male Fischer 344 rats (Jung et al. 2004; Akintola et al. 2008). Given that the cadherin/catenin complex may regulate both proliferation and migration (Watabe et al. 1994; Vasioukhin et al. 2001; Lien et al. 2006; Shih and Yamada 2012; Cui and Yamada 2013), we hypothesized that the loss of α‐catenin would decrease repair in tubular epithelial cells. In the current study, we generated at a cell line with stable knockdown of α(E)‐catenin (C2 cells) and investigated the wound repair phenotype.

## Materials and Methods

### Animals

Male Fisher 344 (F344) rats were obtained from the NIA colony. On the day of the experiment, rats were anesthetized by ketamine (80–120 mg/kg)/xylazine (5–10 mg/kg) intraperitoneal (IP) injection. Kidneys were collected and 1‐mm cross sections were snap frozen in liquid nitrogen and/or embedded in Tissue‐Tek OCT compound (Andwin Scientific, Schaumburg, IL) and frozen. The remaining kidney tissue was fixed in 4% paraformaldehyde overnight and stored in 70% EtOH. All experimental procedures and animal care were approved by the University of Missouri Animal Care and Use Committee in accordance with the NIH.

Flash‐frozen kidney tissue isolated from nonhuman primate samples was obtained from the NIA.

### Western blot

Subconfluent cells were washed twice with ice‐cold phosphate‐buffered saline (PBS, Gibco [Grand Island, NY], Life Technologies, Carlsbad, CA) and lysed with 10 mmol/L Tris‐1% SDS buffer with Halt^TM^ Protease/Phosphatase inhibitor (Thermo Fisher Scientific, Waltham, MA). Cells were scraped and incubated for 15 min at 4°C on a rocker. Cells were further disrupted by passing through a 20‐gauge needle and spun at 12,000 × g for 15 min at 4°C. Tissue lysates were isolated using a 10 mmol/L Tris‐1% SDS buffer supplemented with Halt™ Protease Inhibitor Cocktail. Protein concentration was determined by the BCA method using a commercially available kit (Thermo Fisher‐Pierce).

The following antibodies were used: anti‐α‐catenin (monoclonal, clone 5), anti‐β‐catenin (monoclonal clone 14), anti‐γ‐catenin (monoclonal clone 15), anti‐p120‐catenin (monoclonal clone 98) and anti‐N‐cadherin (monoclonal clone 32), anti‐E‐cadherin (monoclonal clone 36) and anti‐P‐cadherin (monoclonal clone 56) from BD Biosciences (San Jose, CA), and anti‐β‐actin (monoclonal, clone AC‐74; Sigma‐Aldrich, St. Louis, MO). Goat‐anti‐mouse HRP conjugate (Jackson ImmunoResearch Laboratories, West Grove, PA) was used at 1:20,000 dilution. Blots were developed using West Femto (Thermo Fisher‐Pierce) and imaged using the ChemiDoc imaging system and quantitation performed using the ImageLab 3.0 software (Bio‐Rad, Hercules, CA).

### Cell culture

Cells were grown in DMEM/F12 (1:1) containing L‐Glutamine and Hepes (Gibco) supplemented with 5–10% FBS (Hyclone, Thermo Fisher Scientific)/50 U/mL penicillin, 50 mg/mL Streptomycin (Gibco) and incubated at 37°C with 5% CO_2_. Cells were harvested with TrypLE Express (Gibco) and pelleted at 290 × g for 5 min at room temperature. Cell lines with stable knockdown of α(E)‐catenin were generated by Sigma‐Aldrich in NRK‐52E cells; NT3 cells are the nontargeted control cells and C2 cells are α‐catenin knockdown clonal cell lines as previously described by our laboratory (Nichols et al. 2014). Cells were grown in the presence of 5 *μ*g/mL puromycin (Sigma‐Aldrich) for lentivirus maintenance.

### Aggregation assay

Confluent cultures of NT3 and C2 cells were detached by incubating in 5 mL Moscona's Low Bicarbonate Buffer (MLB)/2.5 mmol/L EDTA for 10 min at room temperature and collected by scraping into a 15 mL tube. Plates were washed in 5 mL MLB, added to the tube, and pelleted. Cells were washed a second time with 5 mL MLB, counted, pelleted, and suspended to a final concentration of 5 × 10^5^ cells/mL in MLB/3 mmol/L CaCl_2_/4 mmol/L MgCl_2_. Twenty‐four‐well plates were pretreated with 500 *μ*L MLB/1% BSA for 20 min and air‐dried 20–30 min. One hundred *μ*L cells (5 × 10^4^) + 100 *μ*L MLB/1% BSA were added to each well for a final concentration of MLB/1.5 mmol/L CaCl_2_/2 mmol/L MgCl_2_/0.5% BSA. In certain experiments, EDTA was added; final concentration of 2 mmol/L. Plates were incubated at 37°C on a rocker platform and visualized at 4× magnification at 0, 3, and 5 h. To quantify aggregation, the area of three to five single cells was determined using the 3‐point circle tool (CellSense software, Center Valley, PA). Single cells and clusters of ≤5 cells were counted individually. The area of larger clusters was measured using the free‐hand polygon tool. Cell number was determined by dividing the area of the clusters by the average area of the three to five single cells. Cells were then sorted into groups of 1–10, 11–50, 51–100, and >100 cells.

### Permeability

Cells (1 × 10^4^ cells/well; 200 *μ*L) were seeded on 24‐well transwell plates (6.5 mm insert; 0.4 *μ*m pore, Corning); 600 *μ*L of media was in the lower chamber. After 24 or 48 h, fresh media (200 *μ*L) containing 0.5 mg/k of FITC‐dextran (Sigma‐Aldrich) were added to upper chamber for 2 h; 100 *μ*L of media was collected from the lower chamber and fluorescence assessed at the 485/528 wavelengths.

### Proliferation

Cells (2 × 10^3^ cells/well, 200 *μ*L) were seeded on 48‐well plates. After allowing to attach for 6 h, cell number was estimated by staining with Janus Green as previously used to estimate cell proliferation (Raspotnig et al. 1999); this value represents T0 for the serum experiment. For study of cell proliferation in serum‐containing media, cells were cultured with 5% FBS and cell proliferation determined over a 120‐h time course. For study of cell proliferation in serum‐free media, cells were established in culture for 24 in 5% FBS (this value represents T0 for the serum‐free experiments); cells were then washed 2× with sterile PBS and cell proliferation determined over a 132 course. There was no difference in attachment rates between C2 and NT3 cells.

### Wound healing assay

Cells were seeded in six‐well plates at 2 × 10^4^ cm^2^ and grown to 80–90% confluence (approximately 24 h). A cross‐scratch was made using a 200 *μ*L pipetman tip. Media were removed and the wells washed 2× with serum‐free media. Cells were then incubated with or without serum plus 5 *μ*g/mL puromycin. To inhibit proliferation, in certain experiments mitomycin C (5 *μ*g/mL, Fisher) was added to serum‐free media plus 5 *μ*g/mL puromycin. Healing was visualized at 4× magnification over 24 h and then stained with Crystal Violet. To quantify healing, the center area of the scratch was measured using the closed polygon tool (CellSense software) and percent closure was determined.

In another experiment, confluent cultures of NRK‐52E cells were allowed to repair for 24 h in DMEM/F12 with or without 5% serum. In certain cultures, 40 *μ*g/mL of GC‐4 (Abcam, Cambridge, MA), a functional inhibitory antibody against N‐cadherin, was added. Cells were fixed with 2% paraformaldehyde and stained with Toluidine blue for visualization.

### Wound healing: RNA isolation

Cells were seeded at 5 × 10^3^ cm^2^ in triplicate on 100‐mm plates and grown to ~80% confluency. Using the wide end of 1000‐*μ*L pipetman tip, two vertical and two horizontal scratches were made, 2–3 mm apart. Plates were washed 2× in serum‐free media then incubated in DMEM/F12 with 4% FBS and 5 *μ*g/mL puromycin until half maximal healing occurred (50% for NT3 at 16 h; 33% for C2 at 22 h). Media were removed and 3 mL PBS added to the plate. Cells along the wound were scraped off and combined to a 50‐mL tube. Plates were washed in 3 mL PBS and added to the tube for samples NT3W (wounded) or C2W (wounded). The remaining cells on the plate were scraped off as above for samples NT3R (rest) or C2R (rest). For the unwounded control, cells were seeded at 2.5 × 10^3^/cm^2^ on a 100‐mm plate and switched to DMEM/F12 with 4% FBS and 5 *μ*g/mL puromycin at the same time as the wounded plates. Cells were harvested at the same time as the wounded plates. After harvest, the cell pellet was suspended in 1 mL PBS and RNA was isolated using EZ Tissue/Cell Total RNA Miniprep Kit with on‐column DNase digestion (cat# R1002; EZ BioResearch, St. Louis, MO) following the kit protocol.

### Real‐time PCR

Cells (5 × 10^6^–1 × 10^7^) were harvested and suspended in 1 mL BPS. Total RNA was isolated using the RNeasy mini kit (cat#74104; Qiagen, Valencia, CA) or EZ Tissue/Cell Total RNA Miniprep Kit (cat# R1002; EZ BioResearch) with on‐column DNase digestion. RNA concentration and quality were determined by spectrophotometry with Nanodrop 2000c (Thermo Scientific) and confirmed by agarose gel electrophoresis. cDNA was generated from 2 *μ*g RNA using the High Capacity cDNA Synthesis Kit (cat# 4368814; Life Technologies, Carlsbad, CA) following the kit protocol. Quantitative PCR was performed in duplicate with 50 ng cDNA/reaction using Taqman assays (Life Technologies, see table) using SsoFast™ Probes Supermix with ROX (Bio‐Rad cat#172‐5251; Hercules, CA) and the CFX96 Touch system (Bio‐Rad) with the following cycling conditions: 95°C for 20 s, then 40 times at 95°C for 1 s, and 60°C for 20 s.

Commercially available TaqMan primer sets were used to assess α(E)‐catenin (Rn01406769_mH), N‐cadherin (Rn00580099_m1), E‐cadherin (Rn00580109‐m1), N‐CAM (Rn00580526_m1), α(N)‐catenin (Rn01421325_m1), α‐catulin (Rn01422644_m1), twist1 (Rn00585470_s1), and Casc3 (Rn00595941_m1) (Life Technologies). For α(T) catenin, quantitative PCR was performed in triplicate with 50 ng cDNA/reaction with SsoAdvanced SYBR Green Supermix (Biorad cat#1725260) with the following cycling conditions: 95°C for 30 s, then 40 times 95°C for 10 s, and 60°C for 30 s followed by a melt curve. Relative quantitation was performed using the Pfaffl method (Pfaffl 2001) normalized to Casc3; the α(T)‐catenin and Casc3 primers are shown in Table 1.

**Table 1.** Primer set for α(T)‐catenin

Gene name	Gene	NCBI Accession number	Primer	Start	Forward primer
α(T) catenin	*Ctnna3*	XM_002725827	Forward	338	5′ GAAGATCTCGAAGCTGCACTCCAG 3′
			Reverse	544	5′ AGATGACACATGCTGCAAGAGGC 3′
Casc3	*Casc3*	NM_147144	Forward	1429	5′ CCTTCATTCCTGCAGCCACGG 3′
			Reverse	1566	5′ CCGCTGGGAAGAGTAACGCTTAG 3′

### Statistics

For the permeability assay, an analysis of variance (ANOVA) was performed followed by *post hoc t*‐tests with the Bonferroni correction to evaluate the statistical significance by the SPSS program; *indicates a significant difference from control NT3 cells (*P* < 0.05). For the aggregation assay, a two‐tailed *t*‐test was performed; *indicates a significant difference from control NT3 cells (*P* < 0.05). In the proliferation experiments, a two‐way ANOVA was performed, *indicates a significant difference from NT3 (*P* < 0.05). For the wound healing experiments, a two‐way analysis of variance was performed, *indicates a significant difference from control nontargeted cells (*P* < 0.05).

## Results

**Figure 1.**
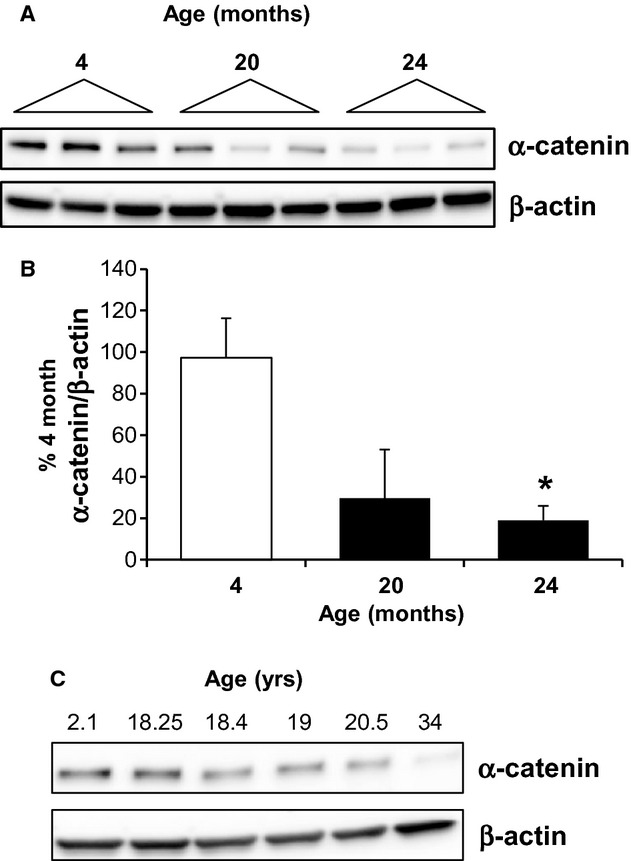
The age‐dependent loss of α‐catenin. (A) Protein expression of α‐catenin was determined by western blot analysis of cortical lysates from male Fischer 344 rats at 4, 20, and 24 months; each lane represents a sample from an individual animal. (B) Densitometric analysis of α‐catenin expression, normalized to β‐actin, is shown; *n* = 3–7 animals, *indicates a significant difference from 4 months. (C) Age‐dependent loss of α‐catenin is also seen in the aging nonhuman primate kidney, as shown by western blot of kidney lysates.

Consistent with previous studies, a decrease in the renal expression of α‐catenin was seen at 24 months in the male Fischer 344 rat model (Fig. 1A and B). A loss of α‐catenin protein expression is also evident at 20 months, suggesting a potential role of decreased α‐catenin in the progression of age‐dependent renal dysfunction. α‐catenin protein expression was also assessed over a time course in aging nonhuman primates. α(E)‐catenin expression was decreased to 60.9% and 15.4% of young (2.1 years) at 20.5 and 34.0 years, suggesting that the age‐dependent decrease in expression was not species specific (Fig. 1C).

**Figure 2.**
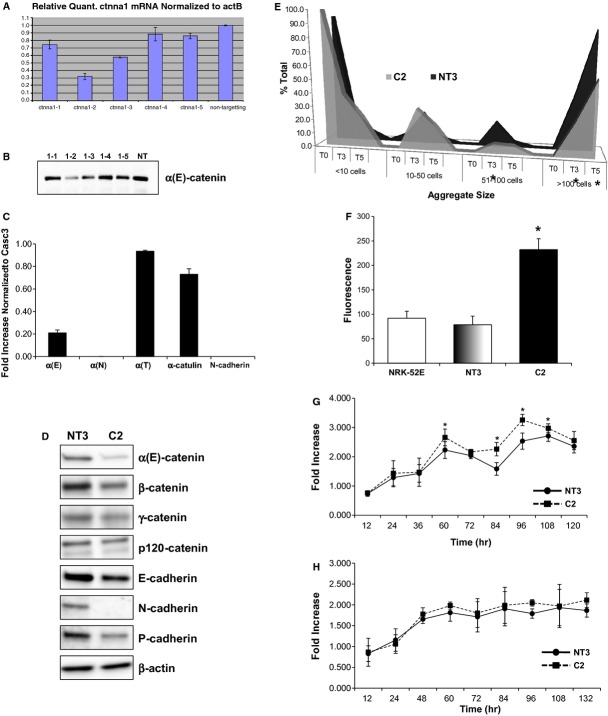
Characterization of stable knockdown of α(E)‐catenin in NRK‐52E cells. Several shRNA constructs targeting α(E)‐catenin were designed and cell lines were commercially generated that demonstrated varying levels of α(E)‐catenin knockdown at the gene (A) and protein level (B). (C) Loss of α(E)‐catenin gene expression in C2 cells is seen as compared to the vector control cell line (NT3). Neither NT3 or C2 cells expressed α(N)‐catenin, and the expression of α(T)‐catenin or α‐catulin was not significantly increased in C2 cells. (D) A corresponding loss of α‐catenin protein expression is seen in C2 cells via western blot analysis. The C2 cells also have decreased β‐catenin and P‐cadherin expression, and almost complete loss of N‐cadherin expression. (E) Analysis of cell aggregation over time demonstrated that a decrease in the number of large cell–cell aggregates (>51 cells/aggregate) in C2 cells as compared to NT3 cells; *indicates a significant difference as compared to NT3 cells. (F) C2 cell monolayers also had increased permeability of FITC‐labeled albumin; *indicates a significant difference in fluorescence as compared to NRK‐52E cells (*n* = 6). Cell proliferation was assessed in serum (G) and serum‐free (H) conditions; each data point represents the fold‐increase over the T0 time point; *n* = 18 from three independent experiments; *indicates a significant difference from NT3.

In an effort to understand the impact of loss of α‐catenin on tubular epithelial cells, a cell line with a stable knockdown of α(E)‐catenin was generated using NRK‐52E cells. Several shRNA constructs targeting α(E)‐catenin were designed and cell lines were generated that demonstrated varying levels of α(E)‐catenin knockdown at the gene and protein level (Fig. 2A and B, respectively). We then generated clonal lines from the parental NT3 (vector control) and 1–2 (knockdown) cells by single cell cloning. The C2 cells are a clonal cell line with significant knockdown of α(E)‐catenin at the gene and protein level (Fig. 2C and D, respectively). C2 cells did not have alterations in expression of α‐catenin‐related genes, including α(N)‐catenin, α(T)‐catenin, or catulin (Fig. 2C). The expression of α(N)‐catenin was not detectable in either C2 or NT3 cells. Gene (Fig. 2C) and protein expression of N‐cadherin, however, was undetectable in C2 cells (Fig. 2D). Protein expression of β‐catenin, and P‐cadherin was also reduced in C2 cells. Decreased expression of cadherin and catenin expression resulted in a loss of cell–cell adhesion in C2 cells (Fig. 2E). Specifically, decreased numbers of large cell aggregates (>51 cells) were seen at 3 and 5 h in the aggregation assay. The loss of α‐catenin expression was also associated with an increase in permeability to FITC‐mannitol (Fig. 2F). Importantly, C2 cells were not characterized by decreased cell proliferation in serum (Fig. 2G) or serum‐free conditions (Fig. 2H). These date indicate that loss of α‐catenin leads to a decrease in function of cadherin/catenin‐mediated cell adhesion in NRK‐52E cells.

**Figure 3.**
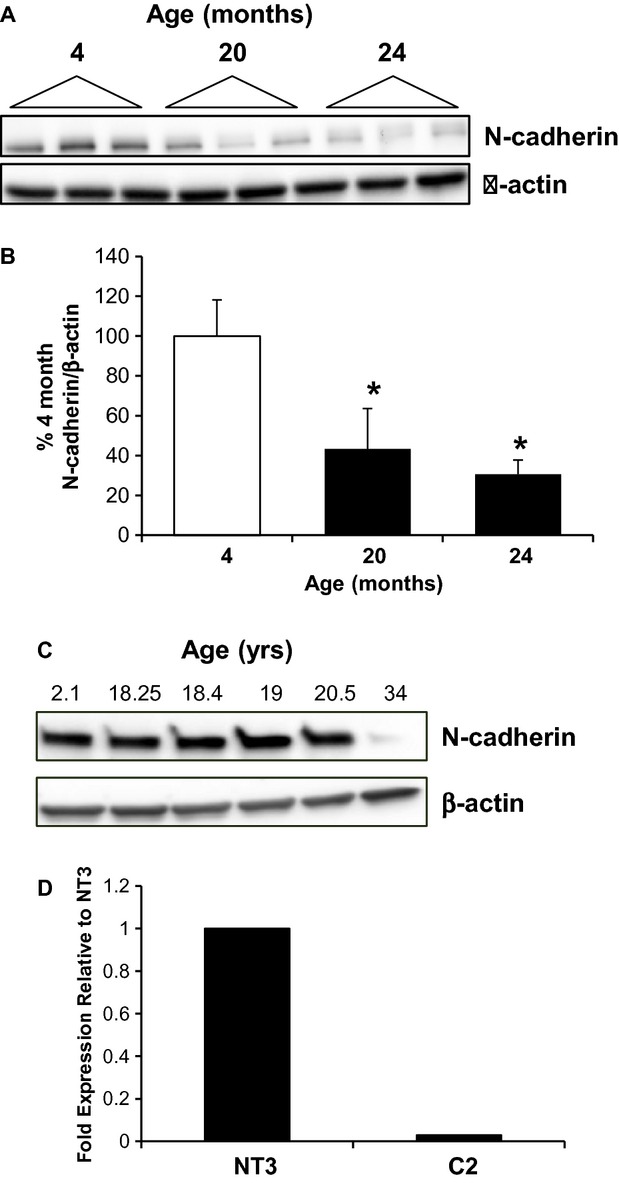
Loss of α‐catenin expression decreases N‐cadherin expression. (A) A loss of N‐cadherin protein expression in cortical lysates from male Fischer 344 rats at 20, or 24 months is shown; each lane represents a sample from an individual animal. (B) Densitometric analysis of N‐cadherin expression, normalized to β‐actin; *n* = 3–7 animals, *indicates a significant difference from 4 months. (C) The age‐dependent loss of N‐cadherin is also seen in the aging nonhuman primate kidney, as shown by western blot of kidney lysates in the bottom panel. (D) The expression of Twist1 is decreased in C2 cells as compared to NT3 controls; similar results were seen in independent experiments.

An age‐dependent loss in protein expression of N‐cadherin in the rat was seen (Fig. 3A and B); interestingly, a linear relationship between the age‐dependent loss of α‐catenin and N‐cadherin is seen, with a correlation coefficient of 0.86. In the nonhuman primate, N‐cadherin expression was stable from 2 to 20.5 years; however, expression was almost undetectable at 34 years (4% of young; Fig. 3C). It is important to note the low number of samples, however, in the nonhuman primate studies. Twist1 is a transcriptional regulator of N‐cadherin (Alexander et al. 2006); interestingly, expression of twist1 is dramatically reduced in C2 cells (Fig. 3D). This suggests that loss of α(E)‐catenin leads to decreased twist1, which could be responsible for decreased N‐cadherin expression. These data suggest that the age‐dependent loss of N‐cadherin that we have previously reported (Jung et al. 2004; Akintola et al. 2008) may be related, in part, to decreased α‐catenin expression.

**Figure 4.**
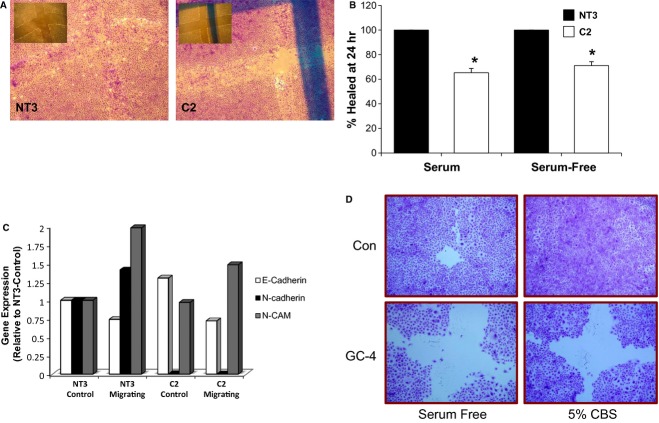
Loss of α(E)‐catenin reduces wound healing. Repair of a scratch in monolayers of C2 cells was inhibited as compared to NT3 cells in serum‐free (SF) conditions. (A) Representative images of NT3 and C2 cells 24 h following injury in serum‐free conditions; the inset in each image is the T0 time point. (B) Quantitative assessment of wound healing at 24 h is shown in both serum and serum‐free conditions; *indicates a statistically significant difference between C2 and NT3, or C2 SF and NT3 SF (*n* = 6). (C) Gene expression of E‐cadherin, N‐cadherin and N‐CAM in control and migrating NT3 and C2 cells. A similar pattern is seen for E‐cadherin and N‐CAM in migrating NT3 and C2 cells, that is, a decrease in E‐cadherin and increase in N‐CAM in both cells. However, migrating NT3 cells exhibit an increase in N‐cadherin gene expression that is not seen in C2 cells. (D) Inhibition of N‐cadherin via a blocking antibody (GC‐4) inhibits repair at 24 h in 5% FBS or serum‐free media.

Wound healing was significantly inhibited in C2 cells in both serum and serum‐free conditions (Fig. 4A and B). Interestingly, in both conditions, the wound was never completely healed. As the role of cell adhesion molecules in migration has been demonstrated, we examined gene expression in the migrating NT3 and C2 cells to identify specific candidate genes that may be altered in C2 cells. As compared to control cultures of NT3 cells, the gene expression of E‐cadherin was slightly increased, N‐CAM expression was similar, and N‐cadherin expression was lost in C2 cells (Fig. 4C). In the migrating NT3 cells, expression of E‐cadherin was decreased, whereas expression of N‐cadherin and N‐CAM was increased compared to control cultures. In migrating C2 cells, a similar decrease in E‐cadherin expression and increase in N‐CAM expression was seen as compared to NT3 cells, although the induction of N‐CAM expression was not as robust. However, there was no upregulation of N‐cadherin expression. The requirement for N‐cadherin in the repair process is demonstrated by the finding that inhibition of N‐cadherin via the GC‐4 blocking antibody reduced wound healing in NRK‐52E cells in either serum‐containing or serum‐free media (Fig. 4D). These data suggest that the α‐catenin‐dependent loss of N‐cadherin expression may underlie the migration deficits of C2 cells.

## Discussion

Our understanding of α‐catenin function has been mainly limited to its role in cadherin‐mediated cell adhesion; however, there are critical cadherin‐independent roles for α‐catenin. In several tumors the loss of α‐catenin is a stronger prognostic factor for dedifferentiation (Kadowaki et al. 1994) and invasion (Kadowaki et al. 1994; Matsui et al. 1994; Gofuku et al. 1999) as compared to E‐cadherin loss. Additionally, increased sensitivity to growth factors, sustained activation of the ras‐ERK pathway, increased NF‐κB activity, and regulation of actin cytoskeleton dynamics are all regulated by α‐catenin in a cadherin‐independent manner (Costa et al. 1998; Kobielak and Fuchs 2006; Lien et al. 2006; Benjamin and Nelson 2008). The loss of α‐catenin expression leads to increased cell proliferation in tumor cells (Watabe et al. 1994), and after targeted knockout in the mouse epidermis (Vasioukhin et al. 2001) and central nervous system (Lien et al. 2006). Consistent with the role in cadherin‐mediated adhesion, our results demonstrate that knockdown of α(E)‐catenin is associated with decreased cell adhesion, and an increased permeability of an epithelial barrier. Interestingly, loss of α(E)‐catenin was associated with downregulation of N‐cadherin expression and decreased ability to repair in a wound healing assay, due to alterations in cell migration. The downregulation of N‐cadherin could be due to the finding that Twist1, a regulator of N‐cadherin expression (Alexander et al. 2006), is reduced by over 99% in C2 cells. Our results are generally consistent with the phenotype of α(E)‐catenin knockdown reported in MDCK cells. In these studies, α(E)‐catenin knockdown is associated with altered cell morphology and disrupted cell–cell adhesion; however, in the wound healing assay the knockdown cells repaired at the same rate as control cells (Benjamin et al. 2010). We hypothesize that the deficits in migration are due to the loss of N‐cadherin expression. The role for N‐cadherin in cell migration has been established by studies from several cell types, including nerve cone migration (Letourneau et al. 1990), myoblasts (Brand‐Saberi et al. 1996), and breast cancer cells (Hazan et al. 2000). More recently, it has been shown that coordinated migration in 3D matrices requires multicellular clusters (Shih and Yamada 2012; Cui and Yamada 2013). In MDCK cells, a N‐cadherin‐actin linkage was required for “efficient” migration in the 3D matrix (Shih and Yamada 2012). This finding was confirmed in PC‐3 cells and, importantly, the N‐cadherin‐dependent migration of the multicellular clusters is regulated by α‐catenin (Cui and Yamada 2013). While the migration rate was increased following α(E)‐catenin knockdown in MDCK cells, migration was not coordinated and it was suggested that migratory deficit in α(E)‐catenin knockdown cells is due to decreased cadherin‐mediated cell adhesion (Benjamin et al. 2010). Taken together, these results are consistent with our hypothesis that α(E)‐catenin‐mediated loss of N‐cadherin expression is important in repair.

While many studies have focused on tubular epithelial cell proliferation in repair (Bonventre 2003; Schmitt et al. 2008b), migration is a key component of renal repair following injury (Hara‐chikuma and Verkman 2006; Hallman et al. 2008). Given that the C2 cells did not have decreased proliferation, we focused on migration as the underlying deficit in the failure to repair. Additionally, data from our laboratory have shown that the significant difference in wound repair in C2 cells is observed when cell proliferation is blocked, further suggesting the importance of migration in this response (Nichols et al. 2014). A potential role of actin in tubular epithelial migration is supported by the findings that Rho inhibitors decrease repair, due to alterations in proliferation and migration (Anderson et al. 2000), while Rho kinase (ROCK) inhibition stimulates migration (Kroening et al. 2010). Given the role of α‐catenin in the regulation of actin dynamics (Kobielak and Fuchs 2004), future studies will focus on the role of α‐catenin‐mediated alterations of actin in the migration of tubular epithelial cells. The translation of these findings in our in vitro models to the in vivo situation, in particular, the potential contribution of altered migration of surviving tubular epithelial cells to the decreased repair of the aging kidney has not yet been determined.

Acute kidney injury on a background of chronic kidney disease (CKD) is an important clinical problem (Singh et al. 2010; Chawla and Kimmel 2012). In rat models of CKD, including aging (Jung et al. 2004; Akintola et al. 2008), the ZDF‐SHHR model of rapidly progressing chronic renal dysfunction (Jung et al. 2004), and the Ren2 transgenic rat model of fibrosis and proteinuria (Whaley‐Connell et al. 2011, 2012), loss of N‐cadherin and α‐catenin expression has been demonstrated. It has been established by our laboratory (Jiang et al. 2004) and others (Leussink et al. 2001; Prozialeck et al. 2003; Nurnberger et al. 2010) that N‐cadherin expression is reduced following acute injury. Therefore, we hypothesize a two‐hit model of the increased severity of AKI on CKD; (1) the loss of N‐cadherin and α‐catenin during CKD that is exacerbated by (2) further loss following injury, which compromises the ability of the kidney to repair. These findings demonstrate that α(E)‐catenin regulates the expression of N‐cadherin, and subsequent migration‐mediated repair in a proximal tubular epithelial cell line. Given the loss of these proteins in the aging kidney, this could explain, in part, the decreased repair capacity following injury.

## Acknowledgments

The authors contributed the following to the work: Single cell cloning, IF (EA G‐B), Permeability (XW), Adhesion assay, qPCR, Proliferation (ARP), Wound Repair (ARP, LAN), Western Blot (ARP), and manuscript preparation (EA G‐B, LAN, ARP). We acknowledge Rebecca Currie for cell counts and determination of cluster areas in the aggregation assays.

## Conflict of Interest

None declared.
